# Promotive, preventive, and treatment interventions for adolescent mental health in sub-Saharan Africa: A protocol for two scoping reviews including systematic analyses of intervention effectiveness

**DOI:** 10.1371/journal.pone.0279424

**Published:** 2022-12-22

**Authors:** James Steel, Fantacy Twagira, Maaike L. Seekles, Angela I. Obasi

**Affiliations:** Department of International Public Health, Liverpool School of Tropical Medicine, Liverpool, United Kingdom; University of Huddersfield, UNITED KINGDOM

## Abstract

**Introduction:**

The increasing prevalence of mental health difficulties amongst adolescents is a worldwide concern. Poor mental health in adolescence is associated with a range of mental, physical and social problems in later life. In sub-Saharan Africa, limited data suggests that mental disorders amongst adolescents are common. It is important that interventions to address this are rooted in an understanding of the unique local context and culture. Yet, the current use, development, and effectiveness of adolescent mental health interventions in sub-Saharan Africa is unclear. In response, this paper presents a protocol for two scoping reviews that together will examine the available evidence on promotive, preventive, and treatment interventions for adolescent mental health in sub-Saharan Africa.

**Methods and analysis:**

The scoping reviews will follow the five-step methodological framework proposed by Arksey and O’Malley, with further recommendations from the Joanna Briggs Institute. They will review scientific and grey literature published between 2000 and 2021, without restrictions in language or study type. A wide range of sources, including MEDLINE, CINAHL, Global Health, PsychINFO, Cochrane and Google Scholar will be searched. Eligibility screening and data extraction will be done by two independent reviewers, and disagreements resolved by a third reviewer. Data will be summarised in two phases. A narrative synthesis will provide a descriptive profile of all studies included and will explore key concepts related to intervention types, target populations and adaptations to local context. A systematic review element will collate evidence of intervention effectiveness from (cluster) Randomised Controlled Trials.

**Discussion and dissemination:**

To the best of our knowledge, these scoping reviews are the first to synthesise a wide range of available evidence on promotive, preventive and treatment interventions for adolescent mental health in sub-Saharan Africa. The results will be published in peer-reviewed publications and will be presented as an evidence base for future intervention development and implementation.

## Introduction

Mental health is increasingly recognised as a global health and development priority [1], integral to meeting the Sustainable Development Goals [2, 3]. Most mental disorders have their first onset in adolescence, yet often remain undiagnosed into adulthood [4]. Adolescent mental health (AMH) issues are major contributors to the disease burden in adolescents. Self-harm, depressive disorders and anxiety are amongst the top ten leading causes of disability adjusted life years in this age group [5]. Further, mental ill-health in adolescence is linked to multiple, adverse biomedical and social outcomes in later life. These include physical ill-health, educational and vocational underachievement, poor interpersonal relationships, and increased risk-taking behaviour [6, 7]. Adolescence is a critical time for both prevention and treatment of mental ill-health [8] with an estimated 63% of all lifetime mental disorders occurring by the age of 25 [9]. Interventions aimed at promoting the mental wellbeing of adolescents and preventing mental difficulties have shown positive outcomes, such as improved social-emotional skills and reduced symptoms of anxiety and depression [10, 11]. Effective evidence-based treatments exists for a range of mental disorders [12].

Ensuring the mental health of adolescents is particularly important in sub-Saharan Africa (SSA). Currently, around 65% of the SSA population is under 25 years of age [13]. By 2050, 40% of all children and adolescents in the world will be Africans [14]. These adolescents may be exposed to a multitude of risk factors for poor mental health, such as poverty and unemployment [8]. Throughout SSA, prevalence data for adolescent mental health disorders are sparse [15]. A recent systematic literature review found median point prevalence rates of 27% for depression, 30% for anxiety disorders and around 40% for emotional and behavioural problems [16].

Mental health interventions in SSA need to be rooted in an understanding of the regions’ unique context. People in the region often have local explanatory models for mental illness, which include a belief in spiritual or supernatural causes for mental illness [17, 18]. Social factors, such as low mental health literacy and perceived or enacted stigma, are barriers to care [17, 19]. Further, SSA has a shortage in trained mental health staff, resulting in a large treatment gap. The median number of mental health workers in the African region (1.6 mental health workers per 100,000 population) is 40 times lower than in the European region (44.8 mental health workers per 100,000 population) [20]. Consequently, there is a need for task-sharing to non-specialist providers and alternative service delivery, such as incorporating traditional healers, that is less pertinent in high-income countries (HICs) [21].

Interventions developed in HICs may not be appropriate in the SSA context, and evidence of their effectiveness may not generalisable. Yet, the current use, development, and effectiveness of AMH interventions in SSA is unclear. Reviews that included studies from low-and-middle income countries included only randomised controlled trials (RCTs) or quasi-experimental studies [10, 22], and did not present information about how interventions were developed or adapted to the local context. Existing evidence of effectiveness is drawn almost exclusively from studies conducted in HICs [12, 23] or in humanitarian or post-conflict settings [24].

To respond to these considerations and gaps, we propose to explore the extent of research related to AMH interventions in SSA. This paper presents a protocol for two scoping reviews on AMH promotive/preventive interventions (review 1) and treatment interventions (review 2). The decision was made to undertake two reviews as opposed to one, to account for expected heterogeneity of included studies and a likely difference in implementation settings and interested audiences. Both reviews will examine and synthesise literature from all study types, to explore the range of interventions used, with a specific focus on adaptations to the local context. An embedded systematic review aspect will collate evidence from (cluster) RCTs on intervention effectiveness.

A preliminary search of MEDLINE and the Cochrane Database of Systematic Reviews was conducted and no current or underway systematic or scoping reviews on the topic were identified.

## Methods

Two scoping reviews will be conducted in accordance with the five-step methodology suggested by Arksey and O’Malley [25] and additional recommendations from Levac et al. [26] and the JBI Manual for Evidence Synthesis [27, 28]. The development of this protocol has drawn on the Preferred Reporting Items for Systematic reviews and Meta-Analysis extension for Scoping Reviews (PRISMA-ScR) checklist [29] (S1 File), which will also be used throughout the review process and in reporting of the findings. A registration on the Open Science Framework has been created for this work (osf.io/3h5ws).

### Stage 1: Identifying the research question

In line with Arksey and O’Malley’s emphasis on the importance of breadth in the scope of the research question and the need for iterative refinement in response to increasing familiarity with the literature [25], the following overarching research questions have been developed:

- Review 1:

“What is known from the existing literature about mental health *promotive or preventive interventions* for adolescents in sub-Saharan Africa?”

- Review 2:

“What is known from the existing literature about *treatment interventions* for adolescent mental ill health in sub-Saharan Africa?”.

For both reviews, sub-questions are likely to include:

- “What are the types of AMH promotion/prevention (review 1) or treatment (review 2) interventions?” and
- “How do these interventions take into account the local context?”
- “What evidence is there of the effectiveness of these interventions?” (Systematic review element)

### Stage 2: Identifying the relevant studies

#### Types of sources and study designs

Both scoping reviews aim to identify published and unpublished material. The databases to be searched are PsychINFO and EBSCOhost, which includes Global Health, MEDLINE complete and CINAHL complete. The Cochrane Library will also be searched. To prevent publication bias, Google advanced search and the British Library EThOS will be used to identify relevant grey literature. In addition, the reference list of key sources of evidence will be screened for additional studies. All study designs will be considered, including qualitative studies and text and opinion papers. Literature reviews will not be included, however these will be examined to identify additional papers that meet the inclusion criteria.

#### Search strategy

In consultation with a librarian, an initial limited search of MEDLINE and CINAHL was undertaken to identify articles on the topic. The text words in the titles and abstracts of relevant articles, and the index terms used to describe the articles were then used to develop full search strategies (see S2 File for example MEDLINE strategies for both reviews). These search strategies, including all identified keywords and subject headings, will be adapted for each database or information source. To ensure that the final reviews contain the most up to date publications possible, search alerts will be set up. The field of global mental health has seen much development since the publication of the Lancet series on global mental health in 2007, which sought to focus the global health spotlight on mental health conditions. To capture an expected increase in studies since this time, whilst still providing an overview of relatively recent evidence, the reviews will include studies published since 2000, in any language. The database searches will be completed by FT for review 1 and JS for review 2.

### Stage 3: Study selection

Following the search, all identified citations will be collated and uploaded into EndNote20 [30] and duplicates removed. Then, all unique citations will be imported into Rayyan software [31] for screening and selection. After a pilot test, titles and abstracts will be screened by two independent reviewers (JS & FT) against the inclusion criteria [26, 27]. Potentially relevant sources will be retrieved in full. The full text will be assessed in detail against the inclusion criteria by two independent reviewers (JS & FT). Reasons for exclusion at full text will be recorded and reported in the scoping review. Disagreements between the reviewers will be resolved through discussion, or by arbitration of a third independent reviewer (MS). The results of the search and the study inclusion process will be reported in full in the final scoping review and documented in a PRISMA-ScR flow diagram [29].

#### Eligibility criteria

The development of pre-defined inclusion criteria was guided by the ‘*Population*, *Concept*, *Context*’ framework suggested in the JBI manual for evidence synthesis [27].

*Population*. The population of interest is adolescents. The WHO defines adolescents as those between the ages of 10–19 years [32], yet this definition is contested [33]. Our initial search further showed that some study samples that include adolescents may not be defined by age, but rather by population group (e.g. secondary school students, or first-year University students). Other studies may take a whole population approach, which includes a proportion of adolescents. To account for this variation, inclusion/exclusion based on age of participants will be as follows: If results are stratified by age, and adolescents made up an age group, the paper will be included. If results are not stratified by age, sources of information will be included if those in the 10–19 age group make up at least 75% of participants. Where only mean or median age is reported, this is required to not be higher than 19 years for the study to be included. Articles that focus on interventions targeting exclusively parents or caregivers, without reporting outcomes for adolescents, will be excluded.

*Concept*. Mental health interventions are usually divided into three categories aimed at promotion, prevention, or treatment [21]. Mental health promotion interventions advocate positive mental health and psychosocial wellbeing, whereas preventive strategies usually target individual risk factors [21]. Preventive interventions can be further subdivided into universal, selective, and indicated prevention, depending on their target population. Universal preventive interventions are aimed at the general population, or a whole population of interest, that has not been identified based on risk; selective prevention is targeted at subpopulations identified as being at elevated risk for a disorder (such as those who have experienced trauma, or adolescents living with HIV); indicated prevention targets individuals who are identified as having minimal but detectable symptoms of a mental disorder [8]. Treatment interventions are deliberately administered to promote recovery or remission of a mental disorder, reduction of symptoms, or improvement of functioning [21]. Treatment interventions specifically target persons living with a mental disorder. Review 1 will include articles with a specific focus on mental health promotion, universal and selective preventive interventions. Review 2 will focus on indicated prevention interventions and treatment interventions. Both reviews will specifically focus on psychosocial interventions; papers that describe interventions that are exclusively pharmacological will be excluded. Papers that describe an intervention for substance (mis)use will be excluded, as a scoping review on the subject has recently been published [34].

*Context*. Understanding the specific context and adaptations to context are key to this scoping review. The review will include studies from SSA in all settings, including but not limited to schools or the community. Studies in humanitarian and conflict-based contexts will be excluded, as reviews of AMH interventions in that setting have recently been completed [24, 35].

The inclusion and exclusion criteria are detailed in Table 1. As seen, review 1 and review 2 only differ in their inclusion criteria based on the concept under investigation. These criteria may change as familiarity with the literature increases.

**Table 1:** Inclusion and exclusion criteria.

	Included Review 1	Included Review 2	Excluded
**Study population**	Adolescents (10–19)	Adolescents (10–19)	Studies with less than 75% adolescents Studies with mean/median age >19
**Intervention type**	Promotion Universal prevention Selective prevention Psychosocial	Indicated prevention Treatment Psychosocial	Alcohol (mis)use intervention Exclusively pharmacological
**Context**	Studies in sub-Saharan Africa	Studies in sub-Saharan Africa	Studies in (post-)conflict or humanitarian settings
**Outcome (evaluation studies only)**	Adolescent mental health and wellbeing benefits (indicators of positive and negative mental health)	Adolescent mental health outcome (indicator of negative mental health)	Mental health outcome not reported Outcome for adolescents not reported
**Publication date**	After 1 January 2000	After 1 January 2000	Before 1 January 2000
**Language**	Any language	Any language	-
**Study design**	Any study design	Any study design	Reviews excluded *after* reference search

### Stage 4: Charting the data

In scoping reviews, ‘charting the data’ refers to the extraction of data. Data will be extracted into Microsoft Excel using a tool developed by the research team (see S3 File for draft tools). For each review, data will be extracted by two independent reviewers, who first complete ten articles and then meet to discuss their results to ensure consistency [26]. The extraction tool will capture details about geographical location, participant demographics, type of intervention, setting, intervention delivery, adaptations to local context, targeted outcomes, and other key aspects relevant to the review question. This process is iterative: the draft data extraction tool will be modified and revised as necessary during data extraction [26, 27]. Modifications will be detailed in the scoping review.

### Stage 5: Collating, summarising, and reporting the results

Both scoping reviews aim to present an overview of all material reviewed. First, a narrative synthesis will present an overview of intervention types and characteristics. This will include qualitative and quantitative studies and will be presented according to the key concepts drawn out of the research. Qualitative analysis (i.e. content analysis) will be used to map intervention components and types of adaptations to local context. Where appropriate, descriptive data (e.g. target population, setting) will be presented using figures and tables. Second, (cluster) RCTs will be investigated to systematically analyse data on intervention effectiveness. The systematic review element for both reviews has been registered on PROSPERO (Review 1 ID: CRD42021297293 and Review 2 ID: CRD42021297295). Cohen’s *d* effect size calculations will be used to obtain an indication of treatment benefits and facilitate comparison of intervention effectiveness presented. For review 1, the primary outcomes of interest are mental health and wellbeing benefits. These include indicators of positive mental health, such as self-esteem; self-efficacy; coping skills; resilience, and indicators of negative mental health, such as depression; anxiety and suicidal behaviour. For review 2, the primary outcomes of interest are related to measurements of mental disorders. Following the Diagnostic and Statistical Manual of Mental Disorders (DSM-V), these include mood disorders (such as depression or bipolar disorder), anxiety disorders, personality disorders, psychotic disorders (such as schizophrenia), eating disorders, trauma-related disorders (such as post-traumatic stress disorder) and neurodevelopmental disorders (such as autism and ADHD). However, if identified, this review will also include measurements related to local conceptualisations of disorders. Although Arksey and O’Malley state that critical evaluation of research is not necessarily a core component of a scoping review [25], we agree with others that there is some role for critical appraisal within a scoping review, particularly in regards to assessment of potential bias in evaluation studies [29, 36]. Therefore, for the systematic review element, the methodology of included articles reporting results of RCTs will be assessed using the Cochrane Risk-of-Bias tool [37]. Articles will be graded as either ‘low’, ‘some’ or ‘high’ risk of bias after assessment of trial design, conduct and reporting.

## Discussion

To the best of our knowledge, these scoping reviews are the first to synthesise a wide range of available evidence on adolescent mental health promotive, preventive and treatment interventions in sub-Saharan Africa. The use of Arksey and O’Malley’s methodological framework [25], with adaptations suggested by the Joanna Briggs Institute [27, 28]], will ensure a transparent and replicable review process. Publication bias will be minimised by the inclusion of grey literature and materials in any language, in addition to searches in African sources that may identify material that is not available in major scientific databases. The findings of the reviews will be disseminated through peer-reviewed publications and will be presented to stakeholders in the field of adolescent mental health during events, such as symposia or conferences. It is expected that findings of this review will be a resource to inform planning of future implementation of interventions and research.

## Supporting information

S1 File PRISMA-ScR fillable checklist. (PDF)

S2 File MEDLINE search strategies. (DOCX)

S3 File Draft data extraction tools. (DOCX)

## References

[1] World Health Organization. The WHO special initiative for mental health (2019–2023): universal health coverage for mental health. Geneva: World Health Organization, 2019 2019. Report No.: Contract No.: WHO/MSD/19.1.

[2] Izutsu T, Tsutsumi A, Minas H, Thornicroft G, Patel V, Ito A. Mental health and wellbeing in the Sustainable Development Goals. The Lancet Psychiatry. 2015;2(12):1052–4. doi: 10.1016/S2215-0366(15)00457-5 26613844

[3] Nations United. Transforming our world: The 2030 Agenda for Sustainable Development. 2015 Contract No.: A/RES/70/1.

[4] Kessler RC, Angermeyer M, Anthony JC, de Graaf R, Demyttenaere K, Gasquet I, et al. Lifetime prevalence and age-of-onset distributions of mental disorders in the World Health Organization’s World Mental Health Survey Initiative. 2007. p. 168–76. PMC2174588 18188442

[5] Vos T, Lim SS, Abbafati C, Abbas KM, Abbasi M, Abbasifard M, et al. Global burden of 369 diseases and injuries in 204 countries and territories, 1990–2019: a systematic analysis for the Global Burden of Disease Study 2019. The Lancet. 2020;396(10258):1204–22. doi: 10.1016/S0140-6736(20)30925-9 33069326 PMC7567026

[6] Nyundo A, Manu A, Regan M, Ismail A, Chukwu A, Dessie Y, et al. Factors associated with depressive symptoms and suicidal ideation and behaviours amongst sub-Saharan African adolescents aged 10–19 years: cross-sectional study. Trop Med Int Health. 2020;25(1):54–69. Epub 2019/11/08. doi: 10.1111/tmi.13336 . 31698526

[7] Patel V, Flisher AJ, Hetrick S, McGorry P. Mental health of young people: a global public-health challenge. The Lancet. 2007;369(9569):1302–13. doi: 10.1016/S0140-6736(07)60368-7 17434406

[8] Organization WH. Guidelines on mental health promotive and preventive interventions for adolescents. Geneva: 2020. 33301276

[9] Solmi M, Radua J, Olivola M, Croce E, Soardo L, Salazar de Pablo G, et al. Age at onset of mental disorders worldwide: large-scale meta-analysis of 192 epidemiological studies. Molecular Psychiatry. 2022;27(1):281–95. doi: 10.1038/s41380-021-01161-7 34079068 PMC8960395

[10] Barry MM, Clarke AM, Jenkins R, Patel V. A systematic review of the effectiveness of mental health promotion interventions for young people in low and middle income countries. BMC Public Health. 2013;13(1):835. doi: 10.1186/1471-2458-13-835 24025155 PMC3848687

[11] Clarke AM, Sorgenfrei M, Mulcahy J, Davie P, Friedrich C, McBride T. Adolescent mental health: a systematic review on the effectiveness of school-based interventions. Early Intervention Foundation, 2021.

[12] Das JK, Salam RA, Lassi ZS, Khan MN, Mahmood W, Patel V, et al. Interventions for Adolescent Mental Health: An Overview of Systematic Reviews. Journal of Adolescent Health. 2016;59(4):S49–S60. doi: 10.1016/j.jadohealth.2016.06.020 27664596 PMC5026677

[13] United Nations. World Population Prospects 2019—Volume II: Demographic Profiles: United Nations; 2020.

[14] United Nations Children’s Fund (UNICEF). Generation 2030 Africa 2.0. 2017 ISBN: 978-92-806-4918-5.

[15] Cortina MA, Sodha A, Fazel M, Ramchandani PG. Prevalence of child mental health problems in sub-Saharan Africa: a systematic review. Arch Pediatr Adolesc Med. 2012;166(3):276–81. Epub 2012/03/07. doi: 10.1001/archpediatrics.2011.592 . 22393184

[16] Jörns-Presentati A, Napp A-K, Dessauvagie AS, Stein DJ, Jonker D, Breet E, et al. The prevalence of mental health problems in sub-Saharan adolescents: A systematic review. PloS one. 2021;16(5):e0251689–e. doi: 10.1371/journal.pone.0251689 . 33989357 PMC8121357

[17] Monteiro N. Addressing mental illness in Africa: Global health challenges and local opportunities. Community Psychology in Global Perspective. 2015;1:78–95. doi: 10.1285/i24212113v1i2p78

[18] Ventevogel P, Jordans M, Reis R, de Jong J. Madness or sadness? Local concepts of mental illness in four conflict-affected African communities. Conflict and health. 2013;7(1):3-. doi: 10.1186/1752-1505-7-3 . 23418727 PMC3605182

[19] Kleintjes S, Lund C, Flisher AJ. A situational analysis of child and adolescent mental health services in Ghana, Uganda, South Africa and Zambia. Afr J Psychiatry. 2010;13(2):132–9. Epub 2010/05/18. doi: 10.4314/ajpsy.v13i2.54360 . 20473475

[20] Organization WH. Mental Health Atlas 2020. 2020 Contract No.: Licence: CC BY-NC-SA 3.0 IGO.

[21] Purgato M, Uphoff E, Singh R, Thapa Pachya A, Abdulmalik J, van Ginneken N. Promotion, prevention and treatment interventions for mental health in low- and middle-income countries through a task-shifting approach. Epidemiol Psychiatr Sci. 2020;29:e150. Epub 2020/08/04. doi: 10.1017/S204579602000061X ; PubMed Central PMCID: PMC7458538. 32744223 PMC7458538

[22] Klasen H, Crombag AC. What works where? A systematic review of child and adolescent mental health interventions for low and middle income countries. Soc Psychiatry Psychiatr Epidemiol. 2013;48(4):595–611. Epub 2012/09/11. doi: 10.1007/s00127-012-0566-x . 22961287

[23] Kuosmanen T, Clarke AM, Barry MM. Promoting adolescents’ mental health and wellbeing: evidence synthesis. Journal of Public Mental Health. 2019;18(1):73–83. doi: 10.1108/JPMH-07-2018-0036

[24] Jordans MJD, Pigott H, Tol WA. Interventions for Children Affected by Armed Conflict: a Systematic Review of Mental Health and Psychosocial Support in Low- and Middle-Income Countries. Current Psychiatry Reports. 2016;18(1). doi: 10.1007/s11920-015-0648-z 26769198 PMC4713453

[25] Arksey H O’Malley L. Scoping studies: towards a methodological framework. International Journal of Social Research Methodology. 2005;8(1):19–32. doi: 10.1080/1364557032000119616

[26] Levac D, Colquhoun H, O’Brien KK. Scoping studies: advancing the methodology. Implementation Science. 2010;5(1):69. doi: 10.1186/1748-5908-5-69 20854677 PMC2954944

[27] Peters M, Godfrey C, McInerney P, Munn Z, Tricco A, Khalil H. Chapter 11: Scoping Reviews (2020 Version). JBI Manual for Evidence Synthesis: Joanna Briggs Institute; 2020. Available from: https://synthesismanual.jbi.global.

[28] Peters MDJ, Marnie C, Tricco AC, Pollock D, Munn Z, Alexander L, et al. Updated methodological guidance for the conduct of scoping reviews. JBI Evid Synth. 2020;18(10):2119–26. Epub 2020/10/11. doi: 10.11124/JBIES-20-00167 . 33038124

[29] Tricco AC, Lillie E, Zarin W, O’Brien KK, Colquhoun H, Levac D, et al. PRISMA Extension for Scoping Reviews (PRISMA-ScR): Checklist and Explanation. Annals of Internal Medicine. 2018;169(7):467–73. doi: 10.7326/M18-0850 30178033

[30] The Endnote Team. EndNote. Endnote 20 ed. Philadelphia, PA: Clarivate; 2013.

[31] Ouzzani M, Hammady H, Fedorowicz Z, Elmagarmid A. Rayyan—a web and mobile app for systematic reviews. Systematic Reviews. 2016;5(1):210. doi: 10.1186/s13643-016-0384-4 27919275 PMC5139140

[32] WHO. Adolescent Mental Health: World Health Organization; 2020 [updated 28/09/2020; cited 2020 20th December]. Available from: https://www.who.int/news-room/fact-sheets/detail/adolescent-mental-health.

[33] Sawyer SM, Azzopardi PS, Wickremarathne D, Patton GC. The age of adolescence. The Lancet Child & Adolescent Health. 2018;2(3):223–8. doi: 10.1016/S2352-4642(18)30022-1 30169257

[34] Mupara LM, Tapera R, Selemogwe-Matsetse M, Kehumile JT, Gaogane L, Tsholofelo E, et al. Alcohol and substance use prevention in Africa: systematic scoping review. Journal of Substance Use. 2021:1–17. doi: 10.1080/14659891.2021.1941356

[35] Jordans MJD, Tol WA, Komproe IH, De Jong JVTM. Systematic Review of Evidence and Treatment Approaches: Psychosocial and Mental Health Care for Children in War. Child and Adolescent Mental Health. 2009;14(1):2–14. doi: 10.1111/j.1475-3588.2008.00515.x

[36] Munn Z, Peters MDJ, Stern C, Tufanaru C, McArthur A, Aromataris E. Systematic review or scoping review? Guidance for authors when choosing between a systematic or scoping review approach. BMC Med Res Methodol. 2018;18(1):143. Epub 2018/11/21. doi: 10.1186/s12874-018-0611-x ; PubMed Central PMCID: PMC6245623. 30453902 PMC6245623

[37] Higgins J, Savovic J, Page M, Elbers R, Sterne J. Chapter 8: Assessing risk of bias in a randomized trial. In: JPT H J T, J C, M C, T L, MJ P, et al., editors. Cochrane Handbook for Systematic Reviews of Interventions Version 62 (updated February 2021): Cochrane; 2021.

