# Supplementary material for: Promotive, preventive, and treatment interventions for adolescent mental health in sub-Saharan Africa: A protocol for two scoping reviews including systematic analyses of intervention effectiveness
Source: PLoS One. 2022 Dec 22;17(12):e0279424. doi: 10.1371/journal.pone.0279424 (PMC9778929; doi:10.1371/journal.pone.0279424)
Supplement: S2 File — (DOCX) [file pone.0279424.s002.docx]

**Supplementary file 2: Appendix 2**

*MEDLINE search strategy review 1 promotive and preventive interventions*

| **Search Stage** | **Keywords** | **Subject Headings (MeSH)** | **LIMITS** |
| --- | --- | --- | --- |
| 1 | child* OR adolescen* OR teen* OR juvenile OR “young person” OR “young people” OR “young adult” OR youth | Adolescent OR Young Adult OR Child |  |
| 2 | “mental health” OR “mental health literacy” OR psychological OR psychosocial OR wellbeing OR emotional OR coping OR resilien* OR mental health stigma OR distress | Mental Health OR Stress, Psychological OR Resilience, Psychological OR Social Support |  |
| 3 | promot* OR prevent* OR awareness OR campaign OR training OR education OR intervention | Primary Prevention OR Health Promotion |  |
| 4 | “Sub-Saharan Africa” OR Angola OR Benin OR Botswana OR “Burkina Faso” OR Burundi OR “Cabo Verde” OR Cameroon OR “Central African Republic” OR Chad OR Comoros OR Congo OR “Cote d’Ivoire” OR “Ivory Coast” OR “Democratic Republic of the Congo” OR Djibouti  OR “Equatorial Guinea” OR Eritrea OR Eswatini OR Swaziland OR Ethiopia OR Gabon OR Gambia OR Ghana OR Guinea OR “Guinea-Bissau” OR Kenya OR  Lesotho OR Liberia OR  Madagascar OR Malawi  OR Mali OR Mauritania OR Mauritius OR Mozambique OR Namibia OR Niger OR  Nigeria OR “Republic of the Congo” OR Rwanda  OR “Sao-Tome and Principe” OR Senegal OR Seychelles OR Sierra Leone OR Somalia OR “South Africa” OR “South Sudan” OR Sudan OR  Tanzania OR Togo OR  Uganda OR Zambia OR Zimbabwe OR “Ivory Coast” | Africa South of the Sahara OR Angola OR Benin OR Botswana OR Burkina Faso OR Burundi OR Cabo Verde OR Cameroon OR Central African Republic OR Chad OR Comoros OR Congo OR Cote d’Ivoire OR Democratic Republic of the Congo OR Djibouti  OR Equatorial Guinea OR Eritrea OR Eswatini OR Ethiopia OR Gabon OR Gambia OR Ghana OR Guinea OR Guinea-Bissau OR Kenya OR  Lesotho OR Liberia OR  Madagascar OR Malawi  OR Mali OR Mauritania OR Mauritius OR Mozambique OR Namibia OR Niger OR  Nigeria OR Rwanda  OR Sao-Tome and Principe OR Senegal OR Seychelles OR Sierra Leone OR Somalia OR South Africa OR South Sudan OR Sudan OR  Tanzania OR Togo OR  Uganda OR Zambia OR Zimbabwe |  |
| 5 | 1 AND 2 AND 3 AND 4 |  |  |
| 6 |  |  | English Language |
| 7 |  |  | Year 2000 |

*MEDLINE search strategy review 2, treatment interventions*

| Stage | Keywords | Subject Headings (MeSH) | Limits |
| --- | --- | --- | --- |
| 1 | AB child* OR AB adolescen* OR AB teen* OR AB juvenile OR AB “young person” OR AB “young people” OR AB “young adult” OR AB youth OR TI child* OR TI adolescen* OR TI teen* OR TI juvenile OR TI “young person” OR TI “young people” OR TI “young adult” OR TI youth | (MH “Adolescent”) OR (MH “Young Adult”) OR (MH “Child+”) |  |
| 2 | AB “mental health” OR AB “mental disorder*” OR AB disorder* OR AB “mental illness” OR AB “well being” OR AB “psychological distress” OR AB schizo* OR AB mania OR AB depression OR AB autism OR AB “post-traumatic stress” OR AB anxiety OR AB ADHD OR AB suicide OR AB “self-harm” OR TI “mental health” OR TI “mental disorder*” OR TI disorder* OR TI “mental illness” OR TI “well being” OR TI “psychological distress” OR TI schizo* OR TI mania OR TI depression OR TI autism OR TI “post-traumatic stress” OR TI anxiety OR TI ADHD OR TI suicide OR TI “self-harm” | (MH “Mental Health”) OR (MH “Mental Disorders+”) |  |
| 3 | AB intervention OR AB support OR AB program* OR AB trial OR AB care OR AB outcome OR AB therap* OR AB treat* OR TI intervention OR TI support OR TI program* OR TI trial OR TI care OR TI outcome OR TI therap* OR TI treat* | (MH “Therapeutics+”) OR (MH “Treatment Outcome+”) OR (MH “Crisis Intervention”) OR (MH “Program Evaluation+”) OR (MH “Program Development”) OR (MH “Pilot Projects”) OR (MH “Mental Health Services+”) OR (MH “Community Mental Health Services”) OR (MH “Psychiatric Rehabilitation”) |  |
| 4 | AB “sub-saharan africa” OR AB Angola OR AB Benin OR AB Botswana OR AB “Burkina Faso” OR AB Burundi OR AB “Cabo Verde” OR AB “Cape Verde” OR AB Cameroon OR AB “Central African Republic” OR AB Chad OR AB Comoros OR AB “Democratic Republic of the Congo” OR AB “Congo Kinshasa” OR AB “Congo Brazzaville” OR AB “Republic of Congo” OR AB Congo OR AB “Cote d'Ivoire” OR AB “Ivory Coast” OR AB Djibouti OR AB “Equatorial Guinea” OR AB Eritrea OR AB Eswatini OR AB Ethiopia OR AB Gabon OR AB “The Gambia” OR AB Ghana OR AB Guinea OR AB “Guinea-Bissau” OR AB Kenya OR AB Lesotho OR AB Liberia OR AB Madagascar OR AB Malawi OR AB Mali OR AB Mauritania OR AB Mauritius OR AB Mozambique OR AB Namibia OR AB Niger OR AB Nigeria OR AB Rwanda OR AB “Sao Tome and Principe” OR AB Senegal OR AB Seychelles OR AB “Sierra Leone” OR AB Somalia OR AB “South Africa” OR AB “South Sudan” OR AB Sudan OR AB Tanzania OR AB Togo OR AB Uganda OR AB Zambia OR AB Zimbabwe OR TI “sub-saharan africa” OR TI Angola OR TI Benin OR TI Botswana OR TI “Burkina Faso” OR TI Burundi OR TI “Cabo Verde” OR TI “Cape Verde” OR TI Cameroon OR TI “Central African Republic” OR TI Chad OR TI Comoros OR TI “Democratic Republic of the Congo” OR TI “Congo Kinshasa” OR TI “Congo Brazzaville” OR TI “Republic of Congo” OR TI Congo OR TI “Cote d'Ivoire” OR TI “Ivory Coast” OR TI Djibouti OR TI “Equatorial Guinea” OR TI Eritrea OR TI Eswatini OR TI Ethiopia OR TI Gabon OR TI “The Gambia” OR TI Ghana OR TI Guinea OR TI “Guinea-Bissau” OR TI Kenya OR TI Lesotho OR TI Liberia OR TI Madagascar OR TI Malawi OR TI Mali OR TI Mauritania OR TI Mauritius OR TI Mozambique OR TI Namibia OR TI Niger OR TI Nigeria OR TI Rwanda OR TI “Sao Tome and Principe” OR TI Senegal OR TI Seychelles OR TI “Sierra Leone” OR TI Somalia OR TI “South Africa” OR TI “South Sudan” OR TI Sudan OR TI Tanzania OR TI Togo OR TI Uganda OR TI Zambia OR TI Zimbabwe | (MH “Africa South of the Sahara+”) OR (MH “Angola”) OR (MH “Benin”) OR (MH “Botswana”) OR (MH “Burkina Faso”) OR (MH “Burundi”) OR (MH “Cabo Verde”) OR (MH “Cameroon”) OR (MH “Central African Republic”) OR (MH “Chad”) OR (MH “Comoros”) OR (MH “Democratic Republic of the Congo”) OR (MH “Congo”) OR (MH “Cote d'Ivoire”) OR (MH “Djibouti”) OR (MH “Equatorial Guinea”) OR (MH “Eritrea”) OR (MH “Eswatini”) OR (MH “Ethiopia”) OR (MH “Gabon”) OR (MH “Gambia”) OR (MH “Ghana”) OR (MH “Guinea”) OR (MH “Guinea-Bissau”) OR (MH “Kenya”) OR (MH “Lesotho”) OR (MH “Liberia”) OR (MH “Madagascar”) OR (MH “Malawi”) OR (MH “Mali”) OR (MH “Mauritania”) OR (MH “Mauritius”) OR (MH “Mozambique”) OR (MH “Namibia”) OR (MH “Niger”) OR (MH “Nigeria”) OR (MH “Rwanda”) OR (MH “Sao Tome and Principe”) OR (MH “Senegal”) OR (MH “Seychelles”) OR (MH “Sierra Leone”) OR (MH “Somalia”) OR (MH “South Africa”) OR (MH “South Sudan”) OR (MH “Sudan”) OR (MH “Tanzania”) OR (MH “Togo”) OR (MH “Uganda”) OR (MH “Zambia”) OR (MH “Zimbabwe”) |  |
| 5 | 1 AND 2 AND 3 AND 4 |  |  |
| 6 |  |  | Year 2000 |
