# Supplementary material for: Promotive, preventive, and treatment interventions for adolescent mental health in sub-Saharan Africa: A protocol for two scoping reviews including systematic analyses of intervention effectiveness
Source: PLoS One. 2022 Dec 22;17(12):e0279424. doi: 10.1371/journal.pone.0279424 (PMC9778929; doi:10.1371/journal.pone.0279424)
Supplement: S3 File — (DOCX) [file pone.0279424.s003.docx]

| **Review 1** | **Review 2** |
| --- | --- |
| Author | Author |
| Year of publication | Year of publication |
| Type of publication | Type of publication |
| Geographical Location | Geographical Location |
| Setting | Setting |
| Study design | Study design |
| Type of study (e.g., evaluation or developmental study) | Type of study (e.g., protocol, evaluation or intervention development study) |
| Type of Intervention | Type of intervention |
| Name of intervention | Name of intervention |
| Description of the intervention | Description of intervention |
| Promotion, universal, or targeted prevention | Target population |
| Target population | Mental health condition targeted |
| Age | Age |
| Gender | Gender |
| Type of person delivering intervention | Delivery of the intervention (by who) |
| Cultural Adaptation | Cultural Adaptations |
| Sample size (if applicable) | Sample size (if applicable) |
| Primary outcomes | Outcomes explored |
| Secondary outcomes (if applicable) | Outcome measurement tool |
| Mental health outcomes captured | Main findings / conclusions |
| Outcome measurement tool | Effect size (if applicable) |
| Main findings / conclusions | Linked publications |
| Effect size (if applicable) |  |
| Linked publications |  |

**Supplementary file 3:**

Appendix 3: draft data extraction tools
